# High Uric Acid Ameliorates Indoxyl Sulfate-Induced Endothelial Dysfunction and Is Associated with Lower Mortality among Hemodialysis Patients

**DOI:** 10.3390/toxins9010020

**Published:** 2017-01-06

**Authors:** Wei-Liang Hsu, Szu-Yuan Li, Jia-Sin Liu, Po-Hsun Huang, Shing-Jong Lin, Chih-Cheng Hsu, Yao-Ping Lin, Der-Cherng Tarng

**Affiliations:** 1 Division of Nephrology, Department of Medicine, Taipei Veterans General Hospital, Taipei 11217, Taiwan; lasote521@gmail.com (W.-L.H.); syli@vghtpe.gov.tw (S.-Y.L.); linyp@vghtpe.gov.tw (Y.-P.L.); 2 Faculty of Medicine, National Yang-Ming University, Taipei 11217, Taiwan; huangbs@vghtpe.gov.tw; 3 Institute of Population Health Sciences, National Health Research Institutes, Zhunan 35053, Taiwan; sgazn.tw@gmail.com (J.-S.L.); cch@nhri.org.tw (C.-C.H.); 4 Division of Cardiology, Department of Medicine, Taipei Veterans General Hospital, Taipei 11217, Taiwan; sjlin@vghtpe.gov.tw; 5 Cardiovascular Research Center, National Yang-Ming University, Taipei 11217, Taiwan; 6 Department of Medical Research, Taipei Veterans General Hospital, Taipe 11217, Taiwan; 7 Department of Health Services Administration, China Medical University, Taichung 40402, Taiwan; 8 Department and Institute of Physiology, National Yang-Ming University, Taipei 11217, Taiwan

**Keywords:** cardiovascular mortality, hemodialysis, indoxyl sulfate, uric acid, vascular toxicity

## Abstract

High uric acid (UA) can act as a pro-oxidant in normal physiological conditions; however, emerging evidence is still debatable with regard to the association between high UA and poor outcomes among chronic hemodialysis (HD) patients. In the present study, 27,229 stable prevalent HD patients were enrolled and divided into four groups according to the quartiles of baseline UA concentration, and 5737 died during a median follow-up of 38 months. Multivariate Cox regression analysis showed that a UA level of <6.1 mg/dL was associated with a higher risk of all-cause mortality compared with a UA level of >8.1 mg/dL [HR, 1.20, 95% CI (1.10–1.31)] adjusting for baseline demographic and biochemical parameters. Moreover, a UA level of <6.1 mg/dL was associated with greater risks of cardiovascular mortality [HR, 1.26, 95% CI (1.13–1.41)] and stroke-related mortality [HR, 1.59, 95% CI (1.12–2.25)], respectively. In vitro experiments further showed an increase in oxidative stress and an inhibition nitric oxide synthesis by indoxyl sulfate (IS) in human aortic endothelial cells, which were significantly attenuated by UA in a dose-dependent manner. We concluded that higher UA in serum was associated with lower risk of all-cause and cardiovascular mortality among HD patients probably through its antioxidant property in ameliorating the IS-related vascular toxicity.

## 1. Introduction

Uric acid (UA) is the end product of purine metabolism in humans. Diet and increased consumption of fructose and alcoholic beverages [1] affect the serum concentration of UA. Many epidemiological studies indicate that hyperuricemia increases the risk for hypertension, insulin resistance, and cardiovascular (CV) disease in the general population [2,3]. Investigators have proposed that hyperuricemia-mediated endothelial dysfunction plays a central role in the development and progression of CV disease, and growing evidence suggests that increased serum UA levels lead to endothelial dysfunction and vascular stiffness [4] because high serum UA initiates oxidative stress and reduces nitric oxide synthesis [5,6].

Emerging evidence is still controversial with regard to the relationship of high UA with poor outcomes among chronic hemodialysis (HD) patients. Three studies based on a relatively small sample size found a J-shaped relationship between uric acid and mortality in HD patients [7,8,9]. A multi-center study with larger case number revealed that higher UA concentrations were associated with lower all-cause and CV mortality in HD patients, suggesting a cardioprotective role of UA in this population [10]. The cause of the “paradox” of high UA with low CV risk may be explained in part by the association of uric acid with nutritional status, but an alternative mechanism such as uric acid’s antioxidant property in hemodialysis patients was still suggested by Latif et al. [10].

Increased risk of CV morbidity and mortality is frequently encountered in HD patients. The Framingham risk equation does not capture the full extent of CV risk in HD patients [11]. Instead, non-traditional risk factors, including anemia, oxidative stress and micro-inflammation, appear to play a pivotal role in promoting CV disease [12]. Moreover, endothelial dysfunction has been observed in a uremic environment, indicating the possible role of uremia-related factors. Protein-bound uremic toxins like indoxyl sulfate and p-cresyl sulfate have detrimental effects on the development of CV disease in uremic settings [13,14]. Indoxyl sulfate (IS) has been known to cause endothelial dysfunction by reducing nitric oxide production and increasing oxidative stress [15,16]. 

UA can initiate oxidative stress, but it also exhibits an antioxidant in plasma [17]. Therefore, UA has a complex and apparently paradoxical role in individuals with high oxidative stress and its antioxidant property is considered to be an alternative mechanism regarding the association between serum UA concentration and mortality in HD patients [10]. In the present study, we investigated the association between higher serum UA concentration and lower all-cause and CV mortality in a large, nationwide cohort of chronic HD patients. We further examined the antioxidant properties of high UA against IS-induced endothelial dysfunction in vitro.

## 2. Results

### 2.1. High UA Levels Are Associated with Lower CV and All-Cause Mortality

**Figure 1:**
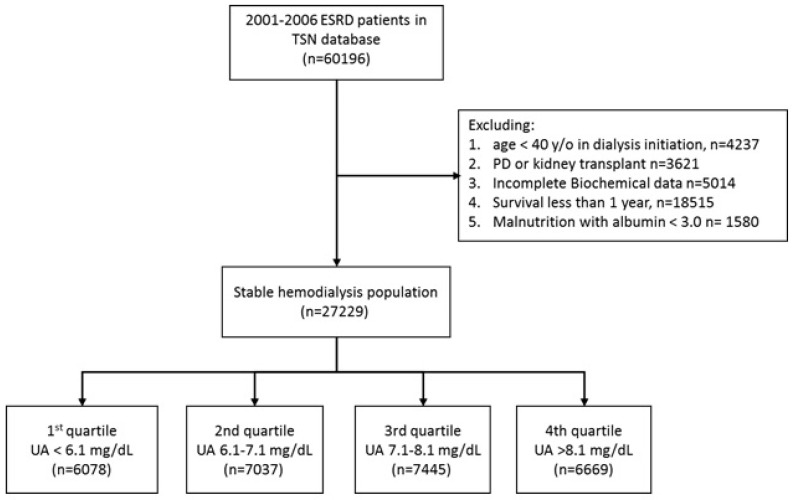
Patient selection flow.

Between 2001 and 2006, a total of 60,196 incident HD patients were registered in the Taiwan Society of Nephrology (TSN) Dialysis Registry. After exclusion of patients for various reasons (Figure 1), 27,229 stable HD patients were enrolled for analysis. We divided these patients into 4 groups according to UA level quartiles. Table 1 shows the baseline demographic data of these 4 groups. The data indicate statistically significant differences among the groups in all measured parameters except serum calcium (Ca). The serum UA level was normally distributed in the study population, with a mean concentration of 7.1 mg/dL (Figure S1).

**Figure 2:**
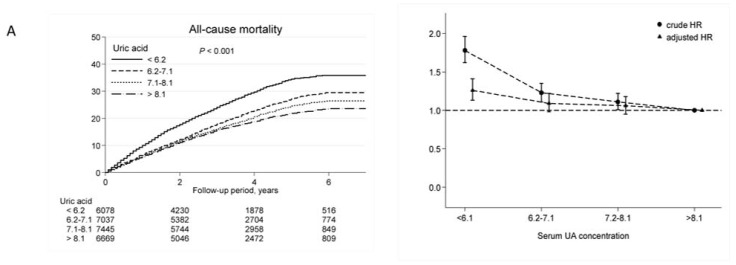

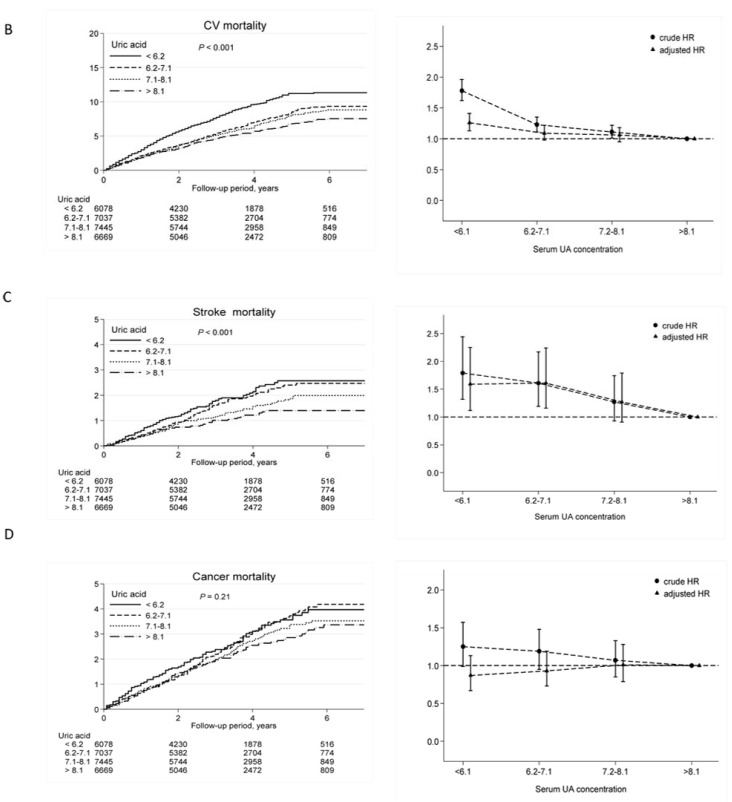
Kaplan–Meier plots (left) and hazard ratios (right) for mortality in chronic hemodialysis patients. Patients with higher UA levels had lower all-cause (**A**), CV-related (**B**), and stroke-related (**C**) mortality, but the cancer mortality (**D**) was comparable among the four UA quartiles. Adjusted hazard ratios (HRs) were calculated using a time-averaged analysis in a multivariate Cox regression analysis.

During a study period of 7 years (median follow-up of 38 months; maximal follow-up of 84 months), 5737 (21%) patients died. Overall, the main causes of death were CV-related (63%), malignant disease (11%), stroke (7%), and others (11%). In multivariate Cox proportional hazard model, the adjusted hazard ratios (aHRs) for all-cause mortality (aHR, 1.20; 95% CI: 1.10–1.31), CV mortality (aHR, 1.26; 95% CI: 1.13–1.41), and stroke mortality (aHR, 1.59; 95% CI: 1.12–2.25) were significantly higher in patients with UA below 6.1 mg/dL (Table 2). However, UA level had no effect on death due to cancer, infection, or GI disorders. Sensitivity tests were performed by analyzing UA in intention-to-treat (first year UA level) and as-treated (last year UA) models, consistent results were obtained (Supplemental Table S1). The results of these separate analyses all indicated a serum UA level below 6.1 mg/dL was associated with higher risk for all-cause and CV mortality in HD patients. Figure 2 shows Kaplan–Meier plots for mortality in patients with different levels of UA (left panel), and the crude HRs and aHRs for mortality in these patients (right panel). These results showed that patients with low UA levels were associated with increased risks of all-cause, CV and stroke mortality, but not cancer mortality.

### 2.2. UA Ameliorates IS-Induced Endothelial Dysfunction In Vitro

**Figure 3:**
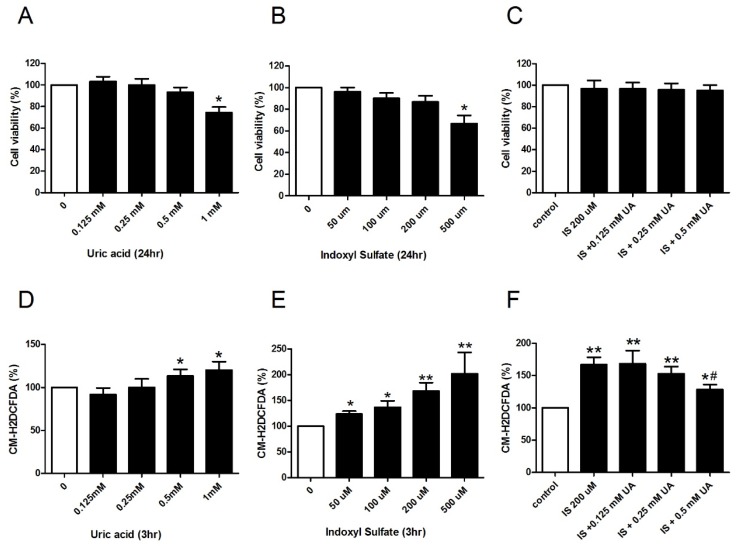

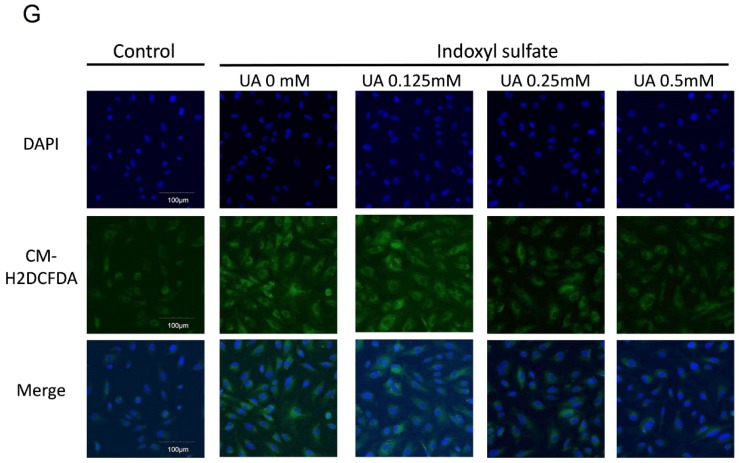
Uric acid attenuates oxidative stress under uremic conditions in vitro. Viability (MTT test) of HAECs following incubation with different concentrations of UA (**A**); indoxyl sulfate (**B**); and 200 µM indoxyl sulfate with different concentrations of UA (**C**); Oxidative stress (CM-H2DCFDA fluorescence) of HAECs following incubation with different concentrations of UA (**D**); indoxyl sulfate (**E**); and 200 µM indoxyl sulfate with different concentrations of UA (**F**); Representative fluorescence microscopy images showing the effect of UA dose (0–0.5 mM) on the attenuation of oxidative stress induced by 200 µM indoxyl sulfate (**G**). * *p* < 0.05 compared to control; ** *p* < 0.01 compared to control; # *p* < 0.05 compared to 200 µM IS. *n* = 6 in each experiment.

We then used an in vitro system to determine whether UA acts as an anti-oxidant in a uremic environment. We used the MTT assay to determine the effect of different IS and UA concentrations on the viability of human aorta endothelial cells (HACEs) (Figure 3). A high concentration of UA (1 mM) or IS (500 µM) decreased the 24-h cell viability. However, simultaneous treatment with low concentrations (0.5 mM UA and 200 µM IS) did not affect HAEC viability. The 200 µM IS used in subsequent experiments is comparable to the IS serum level in uremic patients [18]. A high UA concentration (0.5 mM and 1 mM) slightly increased oxidative damage (Figure 3D), but IS caused oxidative damage at 50 µM (Figure 3E). Interestingly, simultaneous incubation of cells in 0.5 mM UA and 200 µM IS led to less oxidative damage than incubation in 200 µM IS alone (Figure 3F). A representative fluorescence microscopy images is illustrated in Figure 3G.

### 2.3. UA Attenuates IS-Impaired Endothelial NO Production

**Figure 4:**
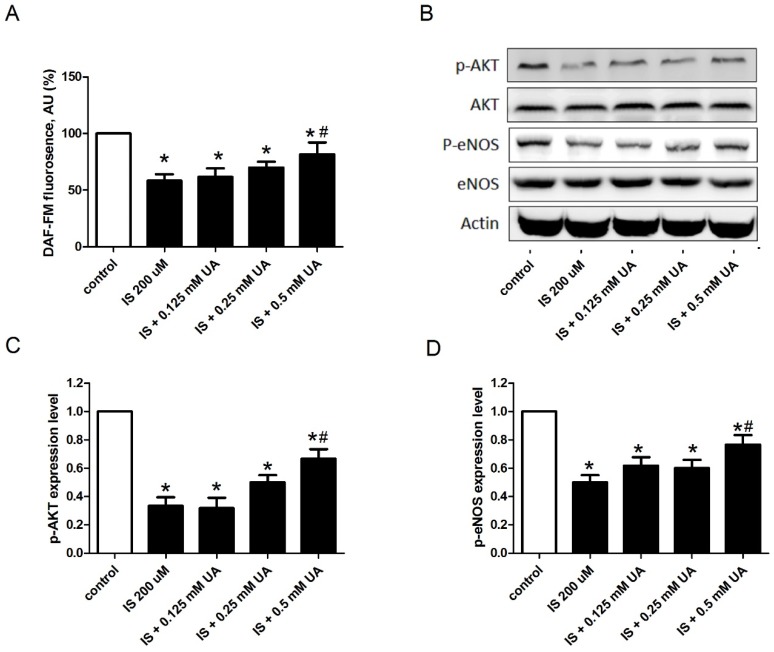
UA preserves endothelial cell NO bioavailability under uremic conditions. Effect of 200 µM indoxyl sulfate with different concentrations of UA on the level of NO (DAF-FM fluorescence) (**A**); and expression of the active and total forms of Akt (**C**) and eNOS (**D**); Panel (**B**) shows representative Western blotting results that are summarized in C and D. * *p* < 0.05 compared to control; # *p* < 0.05 compared to IS 200uM. *n* = 4 in each experiment.

NO, produced by the endothelial NO synthase (eNOS), is fundamental to cardiovascular homeostasis, and an intact Akt-eNOS-NO signal pathway is critical for maintaining endothelial cell function [19,20]. Thus, we determined the effect of IS on the levels of Akt, eNOS, and NO in HAECs (Figure 4). The results indicate that 200 µM IS significantly reduced NO production, but co-incubation of 200 µM IS with 0.5 mM UA partially reversed this effect. The Western blotting results indicate that although 200 µM IS did not influence the levels of Akt and eNOS, it significantly decreased the levels of the activated forms of these enzymes (p-Akt and p-eNOS). Moreover, co-incubation of 200 µM IS with 0.5 mM UA partially reversed this effect.

## 3. Discussion

### 3.1. Main Findings

**Figure 5:**
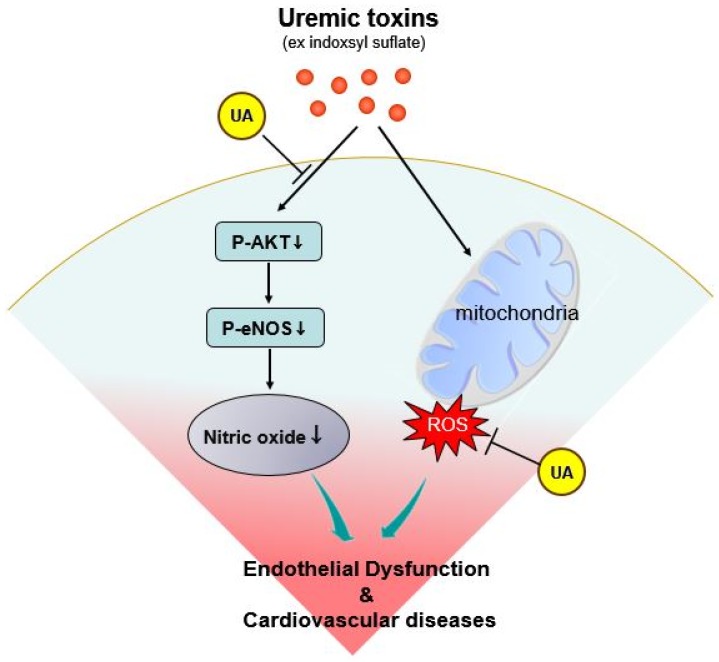
Proposed protective role of UA on the endothelium under uremic condition. End-stage renal disease patients have an extremely high CV mortality rate because uremic toxins induce oxidative stress, but also have decreased nitric oxide production due to inhibition of the Akt-eNOS pathway. These lead to endothelial dysfunction and subsequent complications. A high-normal UA concentration has anti-oxidant effects and preserves NO bioavailability, thereby improving endothelial function and decreasing the risk of CV-related mortality.

In the current study, we used cell culture experiments to demonstrate that a high level of UA (0.5 mM equals to 8.4 mg/dL) attenuates endothelial dysfunction, and the inhibition of IS induced oxidative stress and the preservation of NO production by uric acid is one of the possible mechanisms. In concert with these in vitro experiments, we also found that a lower serum UA concentration is associated with higher risks for all-cause and CV mortality among HD patients. Our data highlight the potentially protective role of UA in vascular toxicity induced by IS in chronic HD patients (Figure 5).

### 3.2. Comparison with Previous Studies

In the general population, there is abundant clinical and experimental evidence that UA contributes to the development and progression of heart and kidney disease because it increases hypertension, activity of the renin-angiotensin-aldosterone system (RAAS), and micro-inflammation, and decreases insulin sensitivity [21,22,23]. However, there is rare evidence that UA-lowering therapy prevents progression of kidney disease or reduces CV events. In fact, a recent double-blind, multicenter, randomized controlled trial of patients with heart failure and moderate CKD found that xanthine oxidase inhibitor did not improve clinical status, quality of life, or left ventricular ejection fraction [24]. Serum UA in the HD population has different prognostic impact from that in general population [10,25]. In HD-dependent patients, some investigators found a J-curve relationship between UA and mortality, while others disclosed an association between high serum UA and lower mortality. The reasons for this discrepancy are probably due to sample size difference, study design, existence of potential confounders, and nutritional status. Our study confirmed the latter association using a nationwide database with a large number of cases.

### 3.3. Potential Mechanisms

There is uncertainty regarding whether UA mediates disease or is only a surrogate of disease severity [26,27]. UA is the end product of purine metabolism in humans, in contrast to most mammals. Thus, due to the loss of uricase activity in the evolution of hominids, primates have higher UA levels than other mammals. Furthermore, 90% of the UA filtered by the kidneys is reabsorbed instead of being excreted. These facts suggest that UA is not simply a harmful waste product, but may be beneficial. Previous studies have interpreted the loss of uricase and the high level of UA in primates as providing an important evolutionary advantage [28]. Oxidative damage to proteins plays a crucial role in aging [29], and UA is one of the most important antioxidants in the human body [30]. Actually, UA is the most abundant aqueous antioxidant, accounting for up to 60% of plasma antioxidative capacity [31]. Therefore, the loss of uricase expression may have provided an evolutionary advantage to hominids [31]. Because UA at physiological concentrations effectively blocks ROS but vitamin C does not [32], the loss of uricase may also compensate for the evolutionary loss of vitamin C synthesis in humans [32,33].

In the current study, UA reduced uremic toxin-induced oxidative stress in endothelial cells. Oxidative stress has emerged as a significant pathogenic feature of uremia. The presence of oxidative stress in ESRD is indicated by an overabundance of the oxidation products of lipids, carbohydrates, and proteins in the plasma and tissues of uremic patients. Uremia has long been interpreted as a sign of premature aging, along with DNA and mitochondrial damage, increased ROS generation, and accelerated vascular disease [34]. Thus, UA may have a beneficial role in uremia, similar to the evolutionary benefit it provides to hominids. A novel finding of the current study is that a high physiological UA concentration preserves NO bioavailability under uremic conditions. Diminished intrinsic NO production and subsequent endothelial dysfunction is a critical problem in uremic patients [35]. Previous experimental studies showed that IS inhibits Akt signaling in various cell types [36,37], and our data confirmed these findings and further linked it to the Akt-eNOS-NO pathway, which is vital for endothelial cell function. Our in vitro experiments clearly illustrated that UA can ameliorate the inhibitory effects of IS and preserve NO bioavailability in HAECs. Our clinical data are consistent with these in vitro experiments, indicating that HD patients with a higher UA level had a lower risk of mortality due to CV and stroke; the risk of mortality from factors unrelated to endothelial cell dysfunction (cancer, GI tract problems, and infection) were not associated with serum UA level.

### 3.4. Limits and Strengths

Compared to previous studies, the current study examined the largest number of HD patients and used in vitro experiments to examine the potential mechanism of the observed clinical effects. We conclude that UA ameliorated IS-induced endothelial damage, and that a high–normal level of UA is beneficial for HD patients. The results of the current study may also have implications for determining the target UA level in dialysis patients, and may influence the prescription of UA-lowering drugs in this population. However, several limitations should be noted. First, the TSN Dialysis Registry does not included data on C-reactive protein, asymmetric dimethylarginine, and NO, and these are important mediators or surrogate markers of endothelial function. Therefore, we cannot correlate serum UA and indoxyl sulfate level with endothelial function in our cohort study. Second, the TSN Dialysis Registry does not include non-fatal events, such as myocardial infarction, heart failure, or gout attack, so we could not examine the relationship of UA level with these events. Among HD patients, uricosuric drugs are not effective, and less than 5% of our HD patients received xanthine oxidase inhibitors. Although xanthine oxidase inhibitors also have anti-oxidant effects [38], we could not meaningfully investigate the association of allopurinol/febuxostat use and survival because the number of patients using these drugs was quite small. Another limitation of this study is that we excluded younger, malnourished patients and those who died within one year after initiation of HD, so our patients might not represent a typical HD population.

## 4. Conclusions

High UA exhibits various activities, from pro-oxidant activities in the general population to anti-oxidant activities in uremic patients. In chronic HD patients, high CV mortality is correlated with endothelial dysfunction partly related to uremic toxins, which exacerbate oxidative stress and reduce NO production. In the current study, we found that a high–normal serum UA concentration can preserve endothelial cell function in vitro and is associated with better survival among chronic HD patients. Therefore, a high–normal UA level in HD patients may be a compensatory mechanism that counteracts the oxidative damage and vascular toxicity from uremic toxins like IS.

## 5. Materials and Methods

### 5.1. Data Sources

TSN Dialysis Registry maintained records of all ESRD patients requiring chronic dialysis in Taiwan [39]. TSN Dialysis Registry database included demographics, disease-associated conditions, dialysis dosage, laboratory data, and clinical outcomes of each dialysis patient in Taiwan. Annual reports of dialysis facilities, treatment quality, and patient information have also been collected. The percentage of reports received from dialysis centers each year has approached 100% since 1997 [39].

### 5.2. Study Participants and Follow Up

To determine the role of UA on the long-term outcomes of HD patients, we reviewed the records of the TSN Dialysis Registry to identify all incident ESRD patients in Taiwan from 1 January 2001 to 31 December 2006. Patients treated by peritoneal dialysis, recipients of kidney transplantation, and those with incomplete biochemistry data were excluded. To avoid survival bias, we excluded patients younger than 40 years old and those who died within one year of initiation of dialysis [40]. To avoid potential bias from malnutrition-related hypouricemia, patients with serum albumin less than 3 g/dL were also excluded. All remaining patients were divided into four groups according to the quartiles of baseline UA level, and were followed up until death or 31 December 2008, whichever came first. Mortality records were retrieved from the Taiwan Death Registry at the Taiwan Ministry of the Interior. The outcome included all-cause mortality, cardiovascular mortality (ICD-9-CM code 410.x, 414.x, 428.x and 440.x), and mortality due to stroke (ICD-9-CM code 433.x, 434.x, or 436) and cancer (ICD-9-CM code 140–208). The institutional review board of Taipei Veterans General Hospital approved the study design. The study was carried out in accordance with the approved protocol and the Declaration of Helsinki.

### 5.3. Cell Culture and Preparation of UA and Indoxyl Sulfate

HAECs were cultured in endothelial growth medium (medium-200 supplemented with LSGS, Life Technologies) with 10% fetal bovine serum (FBS) as described previously [40]. These cells were then incubated with a protein bound IS to mimic endothelial dysfunction in HD patients [35]. UA has very poor water solubility, and re-crystallization can occur at low temperatures. Because of this, UA cannot be prepared in a high-concentration stock solution, but must be freshly prepared at the working concentration. Thus, UA powder (Sigma-Aldrich, St. Louis, MO, USA) was dissolved in 60 °C distilled water in an ultrasonic sonicator for 30 min before experiments; after cooling to 37 °C, the solution was added to the culture medium. Most serum IS exists in a protein-bound form rather than free, so IS powder (Sigma-Aldrich, St. Louis, MO, USA) was mixed with FBS overnight and then added into the culture medium before experiments.

### 5.4. Determining Oxidative Stress And Nitric Oxide In Vitro

A commercially available indicator of general oxidative stress (CM-H2DCFDA) was used. The cell-permeable H2DCFDA (Thermo Scientific Inc., Waltham, MA, USA) passively diffuses into cells and remains within the cell after cleavage by intracellular esterases. Upon oxidation, the non-fluorescent H2DCFDA was converted to the highly fluorescent CM-H2DCFDA, which was better retained within living cells than H2DCFDA. Thus, HACEs cultured in 24-well plates were incubated with 10 µM H2DCFDA for 30 min, washed with sterile PBS, and incubated with UA and IS at different concentrations for 3 h. To determine the effect of UA on IS-induced ROS production, HAECs were pre-treated with different concentrations of UA for 30 min, and then incubated with 200 µM IS for 3 h. The IS incubation time was determined according to previous studies [15,35]. Fluorescence at 527 nm (excitation, 485 nm) was determined with an automatic plate reader.

NO has an extremely short physiological half-life, so special methods are needed to measure the reaction products of NO biochemistry. Thus, we used a standard diaminonaphthalene-based assay to determine NO production according to manufacturer’s protocol [41]. Briefly, HAECs were treated as described above, and loaded with 10 μM of a NO-sensitive fluorescence probe (DAF-FM, Thermo Scientific Inc.) for 30 min. Fluorescence at 515 nm (excitation, 495 nm) was measured with an automatic plate reader. Data are presented as means ± SDs of six independent experiments.

### 5.5. Western Blotting

Proteins in cell lysates were separated by sodium dodecyl sulfate-polyacrylamide gel electrophoresis (SDS-PAGE), transferred to PVDF membranes, and detected by Western blot analysis according to established protocols. The primary antibodies were: anti-phospho-eNOS and anti-eNOS (Abcam, Cambridge, MA, USA), and anti-phospho-AKT and anti-AKT (Cell Signaling Technology, Danvers, MA, USA). Anti-β-actin was used as a loading control. Independent Western blotting experiments were performed four times, and protein expression was quantified by densitometry.

### 5.6. Statistical Analysis

All values were expressed as means and SDs unless otherwise specified. For the in vitro experiments, multiple groups were compared by analysis of variance (ANOVA), with Bonferroni’s post hoc correction. For clinical data, baseline characteristics were compared by ANOVA or chi-square tests. In a multivariable Cox regression model, the effect of UA was adjusted for age, gender, diabetes mellitus, comorbid diseases, cause of ESRD, type of vascular access, dialysis adequacy (Kt/V), normalized protein catabolic rate (nPCR), hematocrit, and serum biochemistry data. Results are expressed as Kaplan-Meier plots or hazard ratios (HRs). A constant HR over time (an assumption of proportional hazards analysis) was evaluated by comparing estimated log-log survival curves for all time-independent covariates. All of these plots had two parallel lines, indicating no violation of this assumption. All *p*-values were two-sided, and the significance level was set at 0.05. All analyses were performed using commercially available software (SAS, version 9.2, SAS Institute Inc., Cary, NC, USA and Stata SE, version 11.0, Stata Corp., College Station, Texas, USA).

## Figures and Tables

**Table 1:** Characteristics of hemodialysis patients with different serum uric acid concentrations.

Parameters	UA Level (mg/dL)				*p* Value
	<6.2	6.2–7.1	7.1–8.1	>8.1	
*n*	6078	7037	7445	6669	
Age, years	66.3 (11.7)	63.5 (11.2)	62.3 (11.2)	61.0 (10.8)	<0.001
Age group, *n* (%)					<0.001
40–64 years	2490 (41)	3596 (51.1)	4164 (55.9)	4049 (60.7)	
65–74 years	1895 (31.2)	2151 (30.6)	2106 (28.3)	1782 (26.7)	
75+ years	1693 (27.9)	1290 (18.3)	1175 (15.8)	838 (12.6)	
Male, *n* (%)	2543 (41.8)	3284 (46.7)	3761 (50.5)	3549 (53.2)	<0.001
DM, *n* (%)	2575 (42.4)	2957 (42)	3050 (41)	2609 (39.1)	<0.001
Kt/V	1.7 (0.3)	1.6 (0.3)	1.6 (0.3)	1.6 (0.3)	<0.001
Hematocrit, %	29.8 (3.3)	30.1 (3.2)	30.2 (3.3)	30.3 (3.6)	<0.001
Ferritin, ng/L	574 (481)	546 (470)	537 (493)	544 (514)	<0.001
TSAT, %	33.2 (14.6)	33.2 (13.6)	32.9 (13.9)	32.3 (14.1)	<0.001
Serum Ca, mg/dl	9.3 (0.8)	9.3 (0.7)	9.3 (0.7)	9.3 (0.8)	0.20
Serum P, mg/dl	4.2 (1.2)	4.7 (1.2)	5.0 (1.2)	5.5 (1.4)	<0.001
Ca*P	33.7 (13.3)	34.3 (15.6)	33.0 (17.9)	29.4 (20.4)	<0.001
iPTH, pg/mL	142 (165)	171 (181)	192 (205)	228 (236)	<0.001

Data are *n* (%), or mean ± SD unless otherwise indicated; Abbreviations: UA: uric acid; DM, diabetes mellitus; TSAT: transferrin saturation; Ca: calcium; P: phosphate; iPTH: intact parathyroid hormone.

**Table 2:** Serum UA level and risk of all-cause, cardiovascular, stroke and cancer mortality among chronic hemodialysis patients.

Uric Acid (mg/dL)	Events	IR	c. HR (95% CI)	a. HR (95% CI)
All-cause mortality				
<6.2	1627	66.3	1.68 (1.56–1.81)	1.20 (1.10–1.31)
6.2–7.1	1511	49.0	1.23 (1.14–1.33)	1.09 (1.01–1.19)
7.1–8.1	1444	43.9	1.10 (1.02–1.19)	1.06 (0.97–1.15)
>8.1	1155	39.8	1.0 (reference)	1.0 (reference)
CV related mortality				
<6.2	1073	43.7	1.78 (1.62–1.96)	1.26 (1.13–1.41)
6.2–7.1	935	30.3	1.23 (1.11–1.35)	1.09 (0.98–1.22)
7.1–8.1	902	27.4	1.11 (1.01–1.22)	1.06 (0.95–1.18)
>8.1	716	24.7	1.0 (reference)	1.0 (reference)
Stroke mortality				
<6.2	101	4.1	1.79 (1.32–2.44)	1.59 (1.12–2.25)
6.2–7.1	115	3.7	1.61 (1.19–2.17)	1.61 (1.16–2.24)
7.1–8.1	97	2.9	1.27 (0.93–1.74)	1.27 (0.91–1.79)
>8.1	67	2.3	1.0 (reference)	1.0 (reference)
Cancer mortality				
<6.2	146	5.9	1.25 (0.99–1.57)	0.87 (0.67–1.13)
6.2–7.1	177	5.7	1.19 (0.95–1.48)	0.93 (0.73–1.19)
7.1–8.1	170	5.2	1.07 (0.85–1.33)	1.01 (0.79–1.28)
>8.1	140	4.8	1.0 (reference)	1.0 (reference)

a. HR: adjusted hazard ratio; CI: confidence interval; c. HR: crude hazard ratio; IR: incidence rate, per 1000 person-years; Cox proportional model was adjusted for age, sex, comorbid disease, diabetes mellitus, Kt/V, hematocrit, ferritin, transferrin saturation, serum albumin, calcium, serum phosphorus, and intact parathyroid hormone.

## Supplementary Materials

The following are available online at www.mdpi.com/2072-6651/9/1/20/s1, Figure S1: Serum UA level in the study population of stable hemodialysis patients. The serum UA level was normally distributed in the study population, with a mean concentration of 7.1 mg/dL (**A**); Analysis of individual patient data indicated that the serum UA concentration changed over time, so the time-averaged UA was not tightly correlated with the first year UA concentration (*r*^2^ = 0.75) or the last year UA concentration (*r*^2^ = 0.77) (**B**,**C**), Table S1: Impact of serum UA level on risk of death by different causes. Hazard ratios were calculated by intention to treat (ITT) and as-treated analysis.
