# Supplementary material for: High Uric Acid Ameliorates Indoxyl Sulfate-Induced Endothelial Dysfunction and Is Associated with Lower Mortality among Hemodialysis Patients
Source: Toxins (Basel). 2017 Jan 6;9(1):20. doi: 10.3390/toxins9010020 (PMC5308252; doi:10.3390/toxins9010020)
Supplement: Supplementary file 1 [file toxins-09-00020-s001.pdf]

Supplementary Materials: High Uric Acid Ameliorates Indoxyl Sulfate-Induced Endothelial Dysfunction and Is Associated with Lower Mortality among Hemodialysis Patients

Wei-Liang Hsu, Szu-Yuan Li, Jia-Sin Liu, Po-Hsun Huang, Shing-Jong Lin, Chih-Cheng Hsu, Yao-Ping Lin and Der-Cherng Tarng

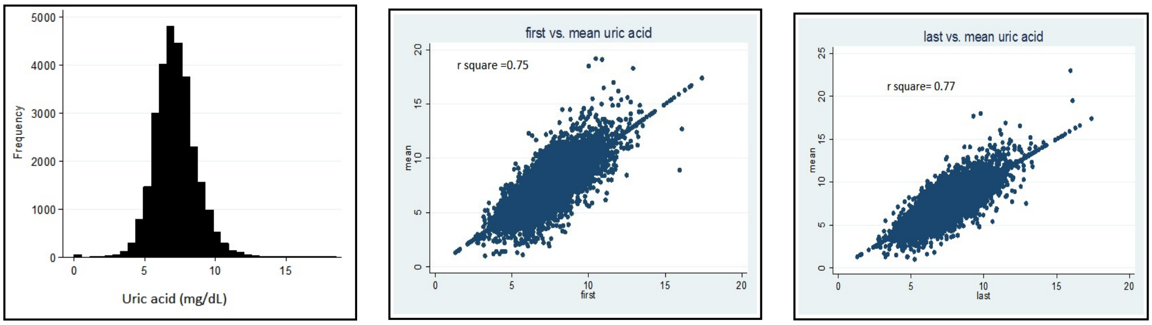

(**a**) (**b**) (**c**)

Figure S1. Serum UA level in the study population of stable hemodialysis patients. The serum UA level was normally distributed in the study population, with a mean concentration of 7.1 mg/dL (**A**); Analysis of individual patient data indicated that the serum UA concentration changed over time, so the time-averaged UA was not tightly correlated with the first year UA concentration (r^2 = 0.75) or the last year UA concentration (r^2 = 0.77) (**B**,**C**).

Table S1. Impact of serum UA level on risk of death by different causes. Hazard ratios were calculated by intention to treat (ITT) and as-treated analysis.

**Uric acid (mg / dL) Subgroups**	**Intention-To Treat (1st Year UA)**		**As-Treated Analysis (Last Year UA)**	
Uric acid	c. HR (95% CI)	a. HR (95% CI)	c. HR (95% CI)	a. HR (95% CI)
All-cause mortality				
<6.2	1.37 (1.28–1.48)	1.10 (1.01–1.20)	1.68 (1.56–1.81)	1.20 (1.10–1.31)
6.2–7.1	1.10 (1.02–1.19)	0.97 (0.88–1.07)	1.23 (1.14–1.33)	1.09 (1.01–1.19)
7.1–8.1	1.05 (0.97–1.13)	1.03 (0.94–1.13)	1.10 (1.02–1.19)	1.06 (0.97–1.15)
>8.1	1.0 (ref.)	1.0 (ref.)	1.0 (ref.)	1.0 (ref.)
CV related mortality				
<6.2	1.40 (1.28–1.53)	1.12 (0.99–1.26)	1.78 (1.62–1.96)	1.26 (1.13–1.41)
6.2–7.1	1.12 (1.02–1.24)	0.99 (0.89–1.12)	1.23 (1.11–1.35)	1.09 (0.98–1.22)
7.1–8.1	1.04 (0.95–1.15)	1.02 (0.91–1.14)	1.11 (1.01–1.22)	1.06 (0.95–1.18)
>8.1	1.0 (ref.)	1.0 (ref.)	1.0 (ref.)	1.0 (ref.)
Stroke mortality				
<6.2	1.67 (1.25–2.23)	1.31 (0.91–1.88)	1.79 (1.32–2.44)	1.59 (1.12–2.25)
6.2–7.1	1.12 (0.82–1.54)	0.96 (0.65–1.41)	1.61 (1.19–2.17)	1.61 (1.16–2.24)
7.1–8.1	1.20 (0.88–1.64)	1.19 (0.83–1.71)	1.27 (0.93–1.74)	1.27 (0.91–1.79)
>8.1	1.0 (ref.)	1.0 (ref.)	1.0 (ref.)	1.0 (ref.)
Infection mortality				
<6.2	1.80 (1.19–2.73)	1.40 (0.81–2.41)	1.95 (1.41–2.71)	1.39 (0.95–2.03)
6.2–7.1	1.15 (0.72–1.82)	0.98 (0.54–1.76)	1.39 (0.99–1.94)	1.29 (0.89–1.87)
7.1–8.1	1.15 (0.73–1.81)	1.28 (0.74–2.22)	1.23 (0.88–1.72)	1.18 (0.81–1.70)
>8.1	1.0 (ref.)	1.0 (ref.)	1.0 (ref.)	1.0 (ref.)
Cancer mortality				
<6.2	1.06 (0.85–1.31)	0.83 (0.64–1.09)	1.25 (0.99–1.57)	0.87 (0.67–1.13)
6.2–7.1	1.01 (0.81–1.26)	0.81 (0.62–1.06)	1.19 (0.95–1.48)	0.93 (0.73–1.19)
7.1–8.1	0.95 (0.76–1.19)	0.85 (0.65–1.11)	1.07 (0.85–1.33)	1.01 (0.79–1.28)
>8.1	1.0 (ref.)	1.0 (ref.)	1.0 (ref.)	1.0 (ref.)

Table S1. *Cont.*

Uric acid (mg / dL) Subgroups	Intention-To Treat (1st Year UA)		As-Treated Analysis (Last Year UA)	
Liver and GI track mortality				
<6.2	1.09 (0.68–1.75)	1.17 (0.65–2.10)	1.71 (1.04–2.83)	1.46 (0.82–2.59)
6.2–7.1	0.94 (0.58–1.55)	0.95 (0.52–1.76)	0.90 (0.52–1.56)	0.88 (0.49–1.6)
7.1–8.1	0.67 (0.39–1.15)	0.90 (0.48–1.67)	0.98 (0.58–1.66)	0.89 (0.50–1.6)
>8.1	1.0 (ref.)	1.0 (ref.)	1.0 (ref.)	1.0 (ref.)
Other cause				
<6.2	1.34 (1.12–1.60)	1.10 (0.88–1.39)	1.41 (1.13–1.76)	0.91 (0.70–1.18)
6.2–7.1	1.05 (0.87–1.28)	1.01 (0.79–1.26)	1.08 (0.87–1.36)	0.92 (0.72–1.17)
7.1–8.1	1.08 (0.89–1.30)	1.15 (0.92–1.44)	0.97 (0.77–1.22)	0.94 (0.74–1.20)
>8.1	1.0 (ref.)	1.0 (ref.)	1.0 (ref.)	1.0 (ref.)

c. HR: crude HR; CI: Confidence interval; a. HR: adjusted HR model with adjusted age, sex, DM, comorbid disease, albumin, Kt/V, hematocrit, ferritin, TSAT, Serum calcium, Serum phosphorus, Ca*P, iPTH and other lab data; CV related: ICD 9 in 250, 410–414, 401–405, 440; Stroke: ICD 9 in 430–438; Cancer: ICD 9 in 140–208; Infection: ICD 9 in 001–139, 320, 321, 322, 326, 420–429, 460–466, 480–487, 490–493,510, 513, 551, 567, 590, 599, 680–686 ,711, 730; Liver, stomach and duodenum: ICD 9 in 531–533, 571.
